# Seasonal influence of tuberculosis diagnosis in Rwanda

**DOI:** 10.1186/s41182-021-00328-w

**Published:** 2021-05-12

**Authors:** Doris Uwamahoro, Aly Beeman, Vinay K. Sharma, Michael B. Henry, Stephanie Chow Garbern, Joseph Becker, Fairuz Despujos Harfouche, Alexis Perez Rogers, Kayla Kendric, Mindi Guptill

**Affiliations:** 1 Department of Anesthesia, Emergency Medicine and Critical Care, University of Rwanda College of Medicine and Health Sciences, Kigali, Rwanda; 2 Department of Emergency Medicine, Warren Alpert School of Medicine, Brown University, Providence, RI USA; 3 Family Medicine Residency Program, Froedtert Hospital Menomonee Falls, Menomonee Falls, WI USA; 4 Columbia University-Vagelos College of Physicians and Surgeons, New York, NY 10032 USA; 5 Department of Emergency Medicine, Maricopa Medical Center-Creighton University Arizona Health Education Alliance, Phoenix, AZ USA; 6 Department of Emergency Medicine, Stanford University School of Medicine, Stanford, CA USA; 7 Department of Emergency Medicine, Loma Linda University School of Medicine, Loma Linda, CA USA

**Keywords:** Tuberculosis, Rwanda, Seasonal influence, Seasonality

## Abstract

**Background:**

Tuberculosis (TB) remains a major global health concern. Previous research reveals that TB may have a seasonal peak during the spring and summer seasons in temperate climates; however, few studies have been conducted in tropical climates. This study evaluates the influence of seasonality on laboratory-confirmed TB diagnosis in Rwanda, a tropical country with two rainy and two dry seasons.

**Methods:**

A retrospective chart review was performed at the University Teaching Hospital-Kigali (CHUK). From January 2016 to December 2017, 2717 CHUK patients with TB laboratory data were included. Data abstracted included patient demographics, season, HIV status, and TB laboratory results (microscopy, GeneXpert, culture). Univariate and multivariable logistic regression (adjusted for age, gender, and HIV status) analyses were performed to assess the association between season and laboratory-confirmed TB diagnoses.

**Results:**

Patients presenting during rainy season periods had a lower odds of laboratory-confirmed TB diagnosis compared to the dry season (aOR=0.78, 95% CI 0.63–0.97, *p*=0.026) when controlling for age group, gender, and HIV status. Males, adults, and people living with HIV were more likely to have laboratory-confirmed TB diagnosis. On average, more people were tested for TB during the rainy season per month compared to the dry season (120.3 vs. 103.3), although this difference was not statistically significant.

**Conclusion:**

In Rwanda, laboratory-confirmed TB case detection shows a seasonal variation with patients having higher odds of TB diagnosis occurring in the dry season. Further research is required to further elucidate this relationship and to delineate the mechanism of season influence on TB diagnosis.

## Background

Tuberculosis (TB) remains a major global health concern with an estimated 10 million new cases in 2019 alone [1]. More than 95% of TB-related deaths occur in low- and middle-income countries (LMICs) such as Rwanda [1]. In 1990, the Rwandan Ministry of Health implemented a six-point “Stop TB Strategy,” and between 1990 and 2013 prevalence and incidence fell by 75% and 76%, respectively [2]. Nonetheless, TB continues to be a major health concern in Rwanda with an incidence of 57/100,000 in 2017 [3].

It has previously been shown that the presentation and diagnosis of many respiratory infections such as TB varies by season. However, most of these studies have been performed done in temperate regions, which are characterized by annual temperature cycles that break the year into four distinct seasons [4–7]. Studies done in temperate regions suggest that the transmission of *Mycobacterium tuberculosis* appears to be the greatest during winter months [7]. This could be due to a variety of factors such as increased indoor activities during these periods, leading to higher transmission and delays in healthcare-seeking behavior [7–9]. Considering that TB has an incubation period which varies greatly from weeks to months, this could explain why symptom onset has been shown to often occur in the months following winter [10, 11]. A systematic review of twelve studies showed a seasonal peak of TB case notifications during the spring and summer seasons [7, 9].

In contrast, Rwanda is located in a tropical equatorial region where seasons are defined by rainfall yielding two distinct seasons, rainy and dry, each occurring twice in varying lengths throughout the year [12, 13]. The few studies that have been conducted on seasonal influence on TB incidence in tropical and equatorial regions have shown conflicting results. One study performed in Cameroon (a Central/West African country with tropical, semi-arid climate) found that more TB cases were diagnosed in the rainy season [14]. However, no seasonal variation was observed in a study from Uganda, while Gashu et al. noted an increase in TB cases during the dry season in Ethiopia [15, 16]. No prior studies have evaluated seasonal variation of TB in Rwanda. A better understanding of TB epidemiology in the context of Rwandan seasonal variability may yield information that could aid in the prevention, control, and management of this disease.

## Methods

### Setting and design

The study was carried out at the University Teaching Hospital-Kigali (French: Centre Hospitalier Universitaire de Kigali, CHUK), the national public referral hospital in Kigali, Rwanda. The facility is an urban, tertiary-care institution with approximately 40 emergency beds and 500 hospital beds. CHUK serves a catchment area of approximately 11 million people and is the national referral center for critically ill patients. A retrospective chart review of patients presenting for care to CHUK with TB laboratory data over a 2-year period (January 2016 to December 2017) was performed.

### Patient population and laboratory data

Patients of any age who presented to either an inpatient or outpatient CHUK facility and who had any laboratory diagnostic test for TB performed were included. The analysis incorporated all types of TB laboratory diagnostics available at CHUK including acid-fast bacilli (AFB) smear microscopy, GeneXpert MTB/RIF (Cepheid Inc., Sunnyvale, CA, USA) polymerase chain reaction (PCR) assay, or cultures. Patients were excluded if they were missing data on age, gender, test results, or date of test. Data abstracted included demographics (age and gender), HIV status (as reported by the patient or from HIV test result as ordered by the treating physician), date laboratory testing was performed, test type, sample type and source, and test result. Dates were categorized by season (rainy or dry). The first dry season was defined as January to February, the first rainy season as March to May, the second dry season as June to August, and the second rainy season as September to December [13].

### Data collection and management

Data were collected from the CHUK paper laboratory records and entered and stored in a password-protected Microsoft Excel database. The study was approved by the CHUK Ethics Committee (EC/CHUK/585/2018). No personally identifiable information was collected.

### Statistical analysis

Descriptive analyses were completed for the overall cohort. Summary statistics were calculated using frequencies and percentages for all categorical variables, and continuous variables were summarized using median values and interquartile ranges. Laboratory-confirmed TB cases were defined as those with any positive TB laboratory test result from microscopy, GeneXpert, and/or culture. The monthly incidence of laboratory-confirmed TB cases and number of patients who had TB laboratory testing performed were calculated and represented graphically. Differences in patient characteristics by season (rainy/dry) were assessed using *χ*^2^ for categorical variables and independent *t* tests for normally distributed continuous variables. A multivariable logistic regression was employed to calculate adjusted odds ratios (aOR) with associated 95% confidence intervals (CIs). The multivariable regression model controlled for age group, gender, and HIV status which were determined a priori through clinical experience and literature review to be associated with TB diagnosis. A significance level of *p* < 0.05 was utilized for all analyses. All statistical analyses were performed using STATA version 15.1 (Stata Corp; College Station, USA).

## Results

**Fig. 1:**
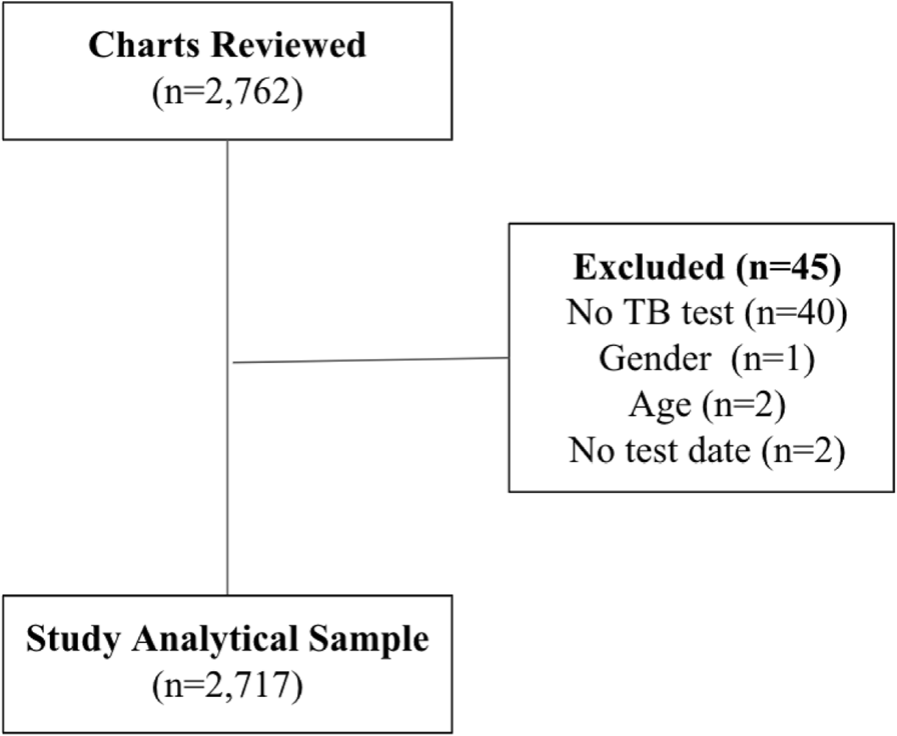
Study population

Of 2762 reviewed patient records, 45 were excluded due to missing data leaving a total of 2717 patients for inclusion in further analysis (Fig. 1). The study population demonstrated a male predominance (58.2%). The median age was 35 years [IQR 22, 52] and 498 (18.3%) were children (< 18 years). Among included patients, 473 (17.4%) had positive HIV test or were self-reported as being HIV positive, 1667 (61.4%) had negative HIV test, and 577 (21.2%) had missing HIV status (no HIV testing performed or no documentation of HIV status), as shown in Table 1.

**Table 1:** Patient characteristics

Characteristic	***n*** (%) (***n***=2717)
**Gender**	
Male	1582 (58.2)
Female	1135 (41.8)
**Age (years),** median [IQR]	35 (22, 52)
**Age group**	
Child (< 18 years)	498 (18.3)
Adult (≥18 years)	2219 (81.7)
**HIV status**	
Negative	1667 (61.4)
Positive	473 (17.4)
Missing	577 (21.2)
**TB status**	
Negative	2237 (82.3)
Positive	480 (17.7)
**Type of test**^a^	
AFB smear microscopy	2289 (84.2)
GeneXpert	1957 (72.0)
Culture	517 (19.0)
**Type of sample**	
Extrapulmonary	869 (32.0)
Pulmonary	430 (15.8)
Unspecified/missing	1419 (52.2)

^a^Variable categories are not mutually exclusive therefore cumulative percentages may be greater than 100%

A total of 4763 laboratory tests (AFB smear microscopy, GeneXpert, culture) for TB were performed during the study period with 480 (17.7%) patients having laboratory-confirmed TB diagnoses (Table 1). The majority (75.0%) of patients had more than one laboratory TB diagnostic test performed. The most common test performed among the study population was an AFB smear microscopy (84.2%), followed by GeneXpert (72.0%) and culture (19.0%). While over half (52.2%) of samples did not explicitly specify the sample source, 869 (32.0%) were documented as being obtained from an extrapulmonary source and 430 (15.8%) from a pulmonary source. The most common sample types specified were sputum (9.8%), cerebrospinal fluid (CSF) (8.0%), and pleural fluid (7.4%), further detailed in Table 2. There was no statistically significant difference between the type of test performed and season (Table 3).

**Table 2:** Sites of TB sampling, pulmonary, or extrapulmonary

Type of testing sample		
Sample type	Specific sample site	***n*** (%) (***n***=2717)
**Pulmonary**	Tracheal aspiration	7 (0.26)
	Bronchoalveolar lavage	156 (5.74)
	Sputum	267 (9.83)
**Extrapulmonary**	Synovial fluid	14 (0.51)
	Cerebrospinal fluid	218 (8.02)
	Ascitic fluid	97 (3.57)
	Urine	66 (2.43)
	Stool	7 (0.26)
	Serum	3 (0.11)
	Gastric aspirate	40 (1.47)
	Pleural fluid	200 (7.36)
	Pus	40 (1.47)
	Tissue/biopsy	165 (6.07)
	Bone marrow	2 (0.07)
	Ocular fluid	1 (0.04)
	Semen	2 (0.07)
**Other**		13 (0.47)
**Missing**		1419 (52.2)

**Table 3:** Type of TB test performed and result by season

	Season		***P***
	Dry	Rainy	
**Gender**			
Male	565 (54.7)	1017 (60.4)	0.003
Female	468 (45.3)	667 (39.6)	
**Age** (mean)	36.49	36.91	0.637
**HIV status**			
Negative	646 (62.5)	1021 (60.6)	0.006
Positive	198 (19.2)	275 (16.3)	
Missing	189 (18.3)	388 (23.0)	
**AFB smear microscopy**	1010	1279	0.537
Negative	905 (89.6)	1156 (90.4)	
Positive	105 (10.4)	123 (9.6)	
**GeneXpert**	705	1252	0.371
Negative	606 (90.0)	1094 (87.4)	
Positive	99 (14.0)	158 (12.6)	
**Culture**	212	305	0.320
Negative	175 (82.5)	247 (81.0)	
Positive	6 (2.8)	17 (5.6)	
Contaminated	31 (14.6)	41 (13.4)	

**Fig. 2:**
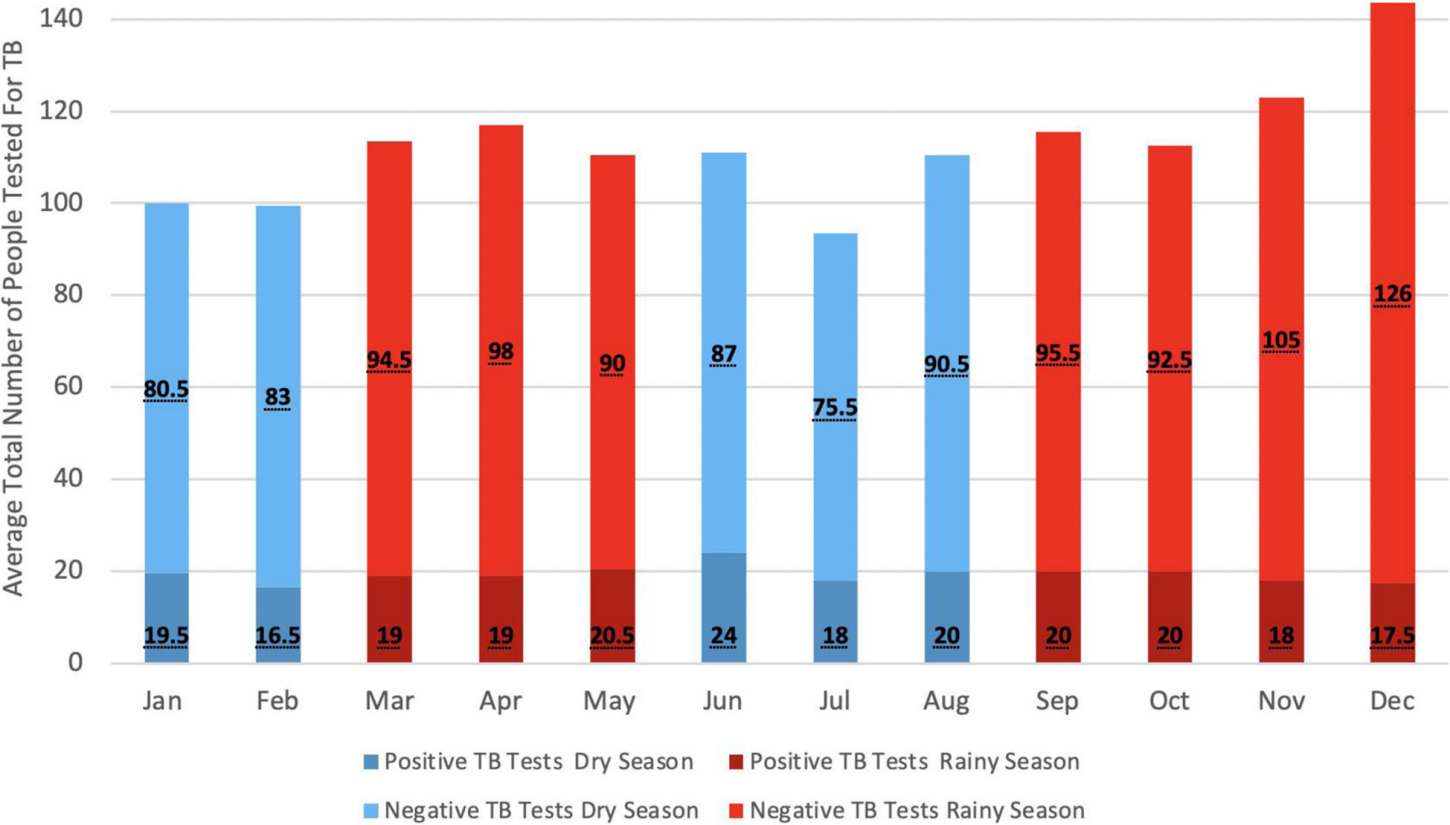
Average number of positive and negative TB tests per month (2016–2017)

The average monthly incidence of patients with laboratory-confirmed TB diagnosis was similar between dry season versus rainy season months (20.1 cases/month vs 19.9 cases/month, respectively, *p*=0.954). There was substantial variation between the two years in terms of monthly incidence of laboratory-confirmed TB. In 2016, July had the lowest incidence of TB diagnoses (6 cases/month), while January and June had the highest incidence (both with 28 cases/month). However, in 2017, January had the lowest incidence of laboratory-confirmed TB (11 cases/month) while October had the highest incidence (33 cases/month) as shown in Fig. 2. The average number of all patients tested per month was higher during the rainy compared to the dry season, although this difference was statistically insignificant (120.3 vs. 103.3 patients/month, respectively, *p*=0.221). There was a significant increase in the number of men tested for TB during the rainy season (60.4%) when compared to the dry season (54.7%) (*p*=0.003).

In unadjusted analysis, females had lower odds of having a positive TB result compared to males (OR=0.81, 95% CI 0.66–0.99, *p*=0.037) and adults had greater odds compared to children (OR=2.05, 95% CI 1.51–2.70, *p*< 0.001). People living with HIV had higher odds (OR=1.40, 95% CI 0.66–0.99, *p*=0.007) of having a positive TB result compared to those HIV negative (Table 4). Patients presenting during a rainy season period had lower odds of positive TB result compared to those in the dry season (OR=0.82, 95% CI 0.68–1.00, *p*=0.055) although was marginally statistically insignificant (Table 4). In multivariable logistic regression analysis, patients who presented during a rainy season period had lower odds of having laboratory-confirmed TB diagnosis than those presenting during a dry season period (aOR=0.78, 95% CI 0.63–0.97), *p*=0.026) when controlling for age group, gender, and HIV status (Table 4).

**Table 4:** Comparison of TB negative and TB positive characteristics

Characteristics	TB negative	TB positive	Univariate		Multivariable	
	***n*** (%) (***n***=2237)	***n*** (%) (***n***=480)	OR (95% CI)	***P***	OR (95% CI)	***P***
**Gender**						
Male (ref)	1282 (57.3)	300 (62.5)	0.81 (0.66–0.99)	**0.037**	0.74 (0.59–0.93)	**0.009**
Female	955 (42.3)	180 (37.5)				
**Age (years),** median [IQR]	35 [22, 52]	35 [25, 50]	1.00 (0.99–1.00)	0.188		
**Age group**						
Child (ref)	446 (19.9)	52 (10.8)	2.05 (1.51–2.78)	**< 0.001**	2.19 (1.55–3.10)	**< 0.001**
Adult	1791 (80.1)	428 (89.2)				
**HIV status**						
Negative (ref)	1365 (61.0)	302 (62.9)	1.40 (1.10–1.79)	**0.007**	1.35 (1.05–1.73)	**0.018**
Positive	361 (16.1)	112 (23.3)				
Missing	511 (22.8)	66 (13.8)				
**Season**						
Dry (ref)	832 (37.2)	201 (41.9)	0.82 (0.67–1.01)	0.055	0.78 (0.63–0.97)	**0.026**
Rainy	1405 (62.8)	279 (58.1)				

## Discussion

Seasonal variation in the diagnosis of TB has been reported in different regions, and various geographic and demographic factors may be involved in seasonality [7, 9]. This study found that laboratory-confirmed TB case detection in a tertiary facility in Rwanda varied by season with lower odds of laboratory-confirmed TB diagnoses during the rainy season compared to the dry season when controlling for age, gender, and HIV status. The delay between transmission of TB (which may occur at higher rates during the rainy season due to greater indoor crowding) to emergence of symptoms weeks or months later and subsequent diagnosis may account for the greater odds of TB case detection in the dry season [9].

A number of studies performed across Africa have shown conflicting results on TB seasonality in tropical climates. Notably, the present study results are similar to what has been observed in Ethiopia, in which a higher case notification rate was observed during the end of dry season [16]. This may be due to the rainy season affecting attendance in health facilities. In contrast, however, our results differ from that of a study completed in Cameroon, which found a higher TB prevalence during the rainy season compared to the dry. This may be explained in that Cameroon has only one distinct rainy and dry season (rainy from April to November, dry from November to March) compared to the two shorter rainy and dry seasons in Rwanda [14]. Due to the long incubation time of TB and insidious initial symptoms, patients in the present study may have been infected with TB during the rainy season but went to seek treatment weeks later when symptoms developed during the dry season, while the longer rainy season in Cameroon may have allowed time for patients to become infected, then develop symptoms and present for care in the same season. Another study performed in Ghana was unable to determine any seasonal pattern between their rainy and dry season [17]. Aryee et al. suggest that it may be due to their lack of information and ability to control for demographic and co-morbid variables (age, gender, HIV status). In the present study, there was no statistical significance between seasons in and laboratory-confirmed TB diagnosis until we controlled for cofounding variables.

Our study demonstrated a trend toward higher testing per month during the rainy season. Although this was not statistically significant, it could potentially suggest a variation in healthcare worker behaviors due to seasonality and warrants further investigation. Reasons for seasonality seem to be more complex and not entirely explained by factors suggested in this and other studies such as healthcare-seeking behavior, crowding during rainy months, vitamin D deficiency [7]. Another possibility could be that Rwanda heavily depends on its agriculture activity. However, Rwanda is prone to suffer considerable food shortages or high food prices rise during the dry season, which could contribute to higher rates of malnutrition which has been associated with higher prevalence’s of TB [18, 19].

While more samples in this study were documented as obtained from extrapulmonary sources than pulmonary, it is likely that the majority of specimens that did not have a source explicitly specified were actually from sputum samples. Prior studies have suggested that specimens for most laboratory-confirmed cases in adults are obtained from pulmonary sources compared to extrapulmonary sources [6].

People living with HIV are 30 times more likely to develop active TB due to impaired immune response [20]. Not surprisingly, this study found a significant association between positive HIV status and laboratory-confirmed TB diagnosis. Due to the retrospective nature of this study, it was not possible to determine which patients were tested for HIV during the same clinical visit as the TB test, or if they had self-reported previously known HIV status to the physician. Those who reported their status may have already been well controlled on HAART, potentially being virally suppressed. Unfortunately, a substantial proportion of patients did not have HIV status documented.

This retrospective study indicated that adults were more likely to be diagnosed with TB compared to children, which is consistent with current literature. A systematic review done in 2011 revealed that TB prevalence in adults increases with age reaching 80% in latent infections at 80 years [7]. Additionally, the challenges of laboratory-confirmation of pediatric TB disease are well documented due to the paucibacillary nature of pediatric disease [21]. Furthermore, we found that males were more likely to have a positive TB result compared to women in unadjusted and adjusted analyses. This finding is consistent with prior studies that have shown that men are disproportionately affected by TB, which could be possibly explained by higher social interactions such as drinking and smoking, or work, which are commonly performed in crowded spaces [7, 13]. However, risk factors and associated behaviors such as health-seeking behaviors for why the incidence of TB diagnosis is higher in men than women are not well understood in the Rwandan context. Further research can help understand these factors and allow for informed public health recommendations targeted to different populations.

## Limitations

Many of the limitations to this study highlight the challenges of conducting research in a resource-constrained setting. First, this was a retrospective study which used pre-existing data that relied on the accuracy of written medical records which were at times incomplete. For instance, as mentioned previously, there was a lack of information on how HIV status was determined for many patients, as well as the source of specimens used for TB testing. Importantly, this study only considered patients with positive TB laboratory test results, as clinically diagnosed TB information was not available; therefore, this study may not accurately reflect actual incidence of TB notifications especially in children in whom clinical diagnosis of TB without confirmatory lab tests is commonplace. In addition, GeneXpert is increasingly being used at CHUK for confirming the diagnosis of TB, while recent studies have found that the sensitivity and specificity of the GeneXpert, specifically in extrapulmonary specimens, varies considerably [22]. Moreover, the data is from an urban tertiary care center and may therefore represent a subset of patients with greater severity of illness. Due to time and resource constraints, it was only feasible to analyze two years of data; seasonal variation may vary substantially between years as seen by the variation between the two years included in this study. There was also some difficulty in defining months as rainy or dry, as there was a lack of consensus in how the seasons are defined, along with annual variations in season onset due to meteorological factors. Finally, due to patient and healthcare worker behaviors and practices, it is difficult to ascertain or speculate the true reasons for the observed greater odds in the dry season.

## Conclusion

This study is the first to report the effects of seasonality on TB diagnosis in Rwanda. Laboratory-confirmed TB detection varied by season, with greater odds of diagnosis during the dry season compared to rainy season. Further research is warranted to understand additional risk factors for TB transmission in the Rwandan population, including the mechanisms by which seasons, and demographic differences influence TB case numbers. Studies should be performed not only at tertiary centers but also at district hospitals to discover more factors that can potentially affect healthcare-seeking behavior (i.e. transportation, socioeconomic status, insurance). Recognizing peak TB seasonality and investigating its context can help with prevention and resource allocation for treatment of TB in Rwanda. Our research could benefit other East African countries with a similar

tropical climate.

## Data Availability

The dataset generated and used during the current study is available from the corresponding author on reasonable request.

## Abbreviations

AFB: Acid-fast bacilli
aOR: Adjusted odds ratios
CHUK: Centre Hospitalier Universitaire de Kigali
CSF: Cerebrospinal fluid
CIs: Confidence intervals
LMICs: Low- and middle-income countries
PCR: Polymerase chain reaction
OR: Odds ratios
TB: Tuberculosis
